# Antimicrobial Susceptibility Pattern of Porcine Respiratory Bacteria in Spain

**DOI:** 10.3390/antibiotics9070402

**Published:** 2020-07-11

**Authors:** Anna Vilaró, Elena Novell, Vicens Enrique-Tarancón, Jordi Balielles, Carles Vilalta, Sonia Martinez, Lorenzo José Fraile Sauce

**Affiliations:** 1 Grup de Sanejament Porcí, 25192 Lleida, Spain; micro@gsplleida.net (A.V.); elena@gsplleida.net (E.N.); Vicens@gsplleida.net (V.E.-T); Jordi@gsplleida.net (J.B.); 2 Freelance researcher, Arbeca, 25192 Lleida, Spain; cvilalta@umn.edu; 3 Departamento de Sanidad Animal, Universidad de León, 24006 León, Spain; smarm@unileon.es; 4 Departament de Ciència Animal, Escola Tècnica Superior d’Enginyeria Agrària, University of Lleida-Agrotecnio, 25198 Lleida, Spain

**Keywords:** antimicrobial susceptibility, swine, respiratory pathogens

## Abstract

The monitoring of antimicrobial susceptibility of pig pathogens is critical to optimize antimicrobial treatments and prevent development of resistance with a one-health approach. The aim of this study was to investigate the antimicrobial susceptibility patterns of swine respiratory pathogens in Spain from 2017 to 2019. Bacterial isolation and identification were carried out following standardized methods from samples coming from sacrificed or recently deceased pigs with acute clinical signs compatible with respiratory tract infections. Minimum inhibitory concentration (MIC) values were determined using the broth microdilution method containing a total of 10 and 7–8 antimicrobials/concentrations respectively, in accordance with the recommendations presented by the Clinical and Laboratory Standards Institute (CLSI). The obtained antimicrobial susceptibility varies between pig respiratory pathogens. *Actinobacillus pleuropneumoniae* (APP) and *Pasteurella multocida* (PM) were highly susceptible (≥90%) to ceftiofur, florfenicol and macrolides (tilmicosin, tildipirosin and tulathromycin). However, the antimicrobial susceptibility was intermediate (>60% but <90%) for amoxicillin and enrofloxacin in the case of APP and sulfamethoxazole/trimethropim and tiamulin in the case of PM. Both bacteria showed low (<60%) antimicrobial susceptibility to doxycycline. Finally, *Bordetella bronchiseptica* was highly susceptible only to tildipirosin and tulathromycin (100%) and its susceptibility for florfenicol was close to 50% and <30% for the rest of the antimicrobial families tested. These results emphasize the need of determining antimicrobial susceptibility in pig respiratory cases in order to optimize the antimicrobial treatment in a case-by-case scenario.

## 1. Introduction

The Porcine Respiratory Disease Complex (PRDC) is a syndrome that results from a combination of infectious and non-infectious factors [1]. *Actinobacillus pleuropneumoniae* (APP), *Pasteurella multocida* (PM), *Mycoplasma hyopneumoniae* (MH), *Bordetella bronchiseptica* (BB) and *Glaesserella (Haemophilus) parasuis* (GP) are the most common bacterial agents involved. Porcine reproductive and respiratory syndrome virus (PRRSV), swine influenza virus (SIV) and porcine circovirus type 2 virus (PCV2) are the most prevalent viral agents [1,2,3,4,5,6]. On the other hand, many non-infectious predisposing factors are also involved in PRDC, such as poor environmental conditions, density, stressors, season of the year, genetic background and production flow (all-in-all out versus continuous flow) [7,8,9]. As a general approach, preventive medicine programs should be based on applying measures to control PRDC in a cost-effective way, such as improving environmental conditions, decreasing density and stressors, combined with vaccination against the major viral and bacterial infectious etiologic factors [10]. However, if such measures are not in place or fail, the use of antimicrobials may be needed.

The use of antimicrobials could be necessary to control bacteria involved in PRDC with a therapeutic or metaphylactic (group medication) goal. In particular, the objective of antimicrobial therapy is to provide an effective drug to obtain a fast, clinical recovery from the infection in affected animals but reducing the probability of generating antimicrobial resistance [11]. However, its use is one of the factors involved in the emergence and spread of bacterial antimicrobial resistance (AR) from pig origin worldwide [12,13]. Resistant bacteria in humans, food, environment and animals are interconnected, and exchange may continuously take place between these ecological niches. For this reason, AR needs to be addressed with a one-health perspective and action plans have been adopted to address this problem [14]. These plans are based on the development of programs to monitor the usage of antimicrobial agents in pig medicine and the occurrence of antimicrobial resistance in pigs at the European level [15,16]. In veterinary medicine, Antimicrobial Susceptibility Testing (AST) data could predict the clinical outcome of antimicrobial treatment, allowing a rational choice of these drugs to treat bacterial infections [11,17]. Antimicrobial susceptibility is usually measured using minimum inhibitory concentration (MIC) that is the lowest antimicrobial concentration that inhibits the growth of the target bacteria in vitro. Moreover, it is necessary to have valid clinical breakpoints (CB) to correctly interpret the MIC value obtained for each clinical case. Thus, all the clinical cases with MIC values below CB could be correctly treated with one antimicrobial at the common registered dose [11]. 

The Spanish national program to control antimicrobial resistance has been adopted to reduce the risk of developing antimicrobial resistance since 2014 [16]. One of the points of the program is focused on reducing the antimicrobial consumption in livestock in order to reduce the prevalence of resistant bacteria. This antimicrobial consumption has been steadily decreasing in the last years according to the available European data [15]. Unfortunately, there is little knowledge of antimicrobial susceptibility patterns for animal pathogenic bacteria in Spain. In this study, we present antimicrobial susceptibility patterns for some of the most important pig respiratory pathogenic bacteria, collected during the period 2017–2019 in Spain. 

## 2. Results

Four-hundred samples were received from sow, wean-to-finish and fattening farms across Spain suffering clinical respiratory cases. In the case of sow farms, the samples were obtained from their nursery facilities. Bacterial isolation (APP, PM and BB) was only possible in 80.3% (321/400) of the cases, and in 22% (88/400) of them, it was possible to isolate more than one bacteria. The isolation of APP and PM and PM and BB was possible in 17.5% (70/400) and 4.5% (18/400) of the cases, respectively.

**Figure 1:**
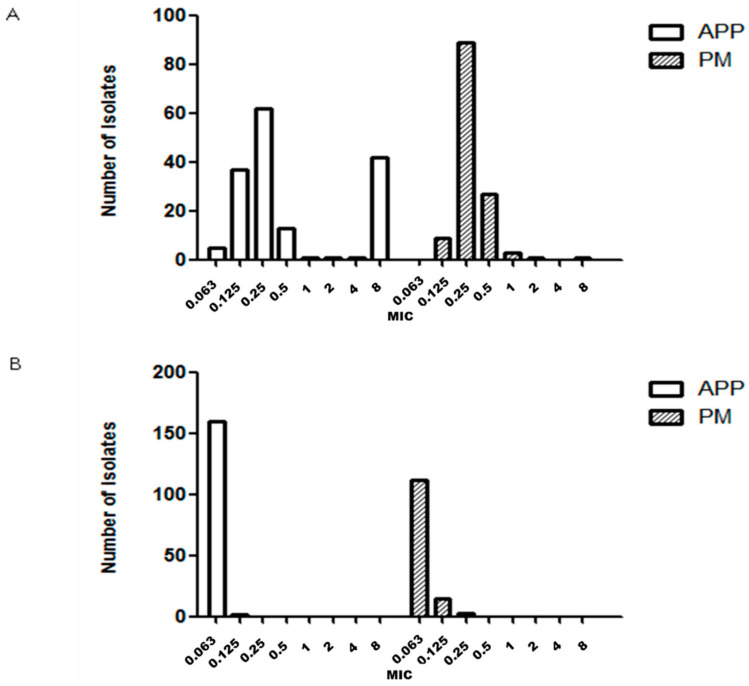
Minimum inhibitory concentration (MIC, μg/mL) distribution of amoxicillin (**A**) and ceftiofur (**B**) for *Actinobacillus pleuropneumoniae* (APP) and *Pasteurella multocida* (PM) isolated from lungs of pigs with respiratory symptoms.

**Figure 2:**
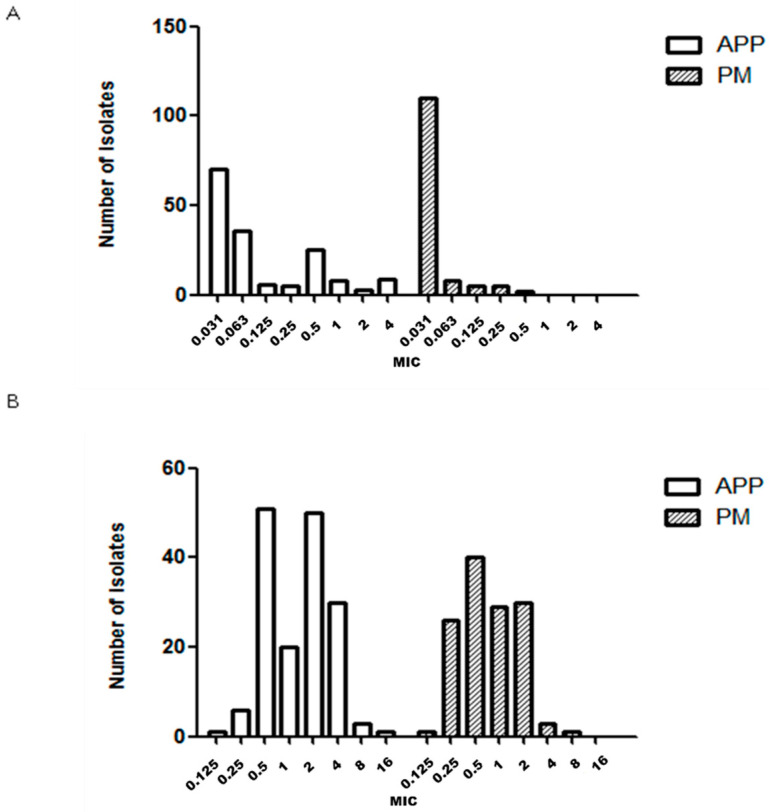
Minimum inhibitory concentration (MIC, μg/mL) distribution of enrofloxacin (**A**) and doxycycline (**B**) for *Actinobacillus pleuropneumoniae* (APP) and *Pasteurella multocida* (PM) isolated from lungs of pigs with respiratory symptoms.

**Figure 3:**
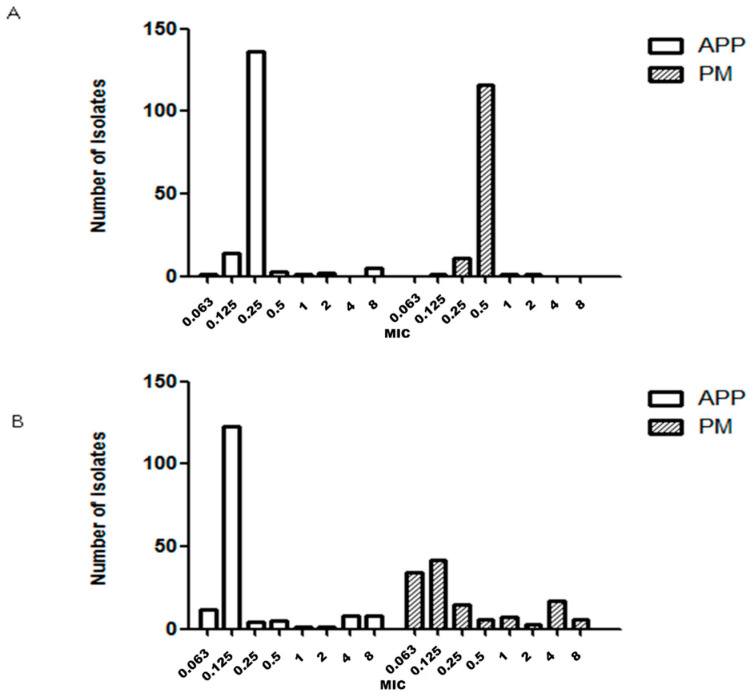
Minimum inhibitory concentration (MIC, μg/mL) distribution of florfenicol (**A**) and sulfamethoxazole/trimethoprim (**B**) for *Actinobacillus pleuropneumoniae* (APP) and *Pasteurella multocida* (PM) isolated from lungs of pigs with respiratory symptoms. In the case of sulfametoxazole/trimethoprim, the MIC value for trimethoprim is represented.

**Figure 4:**
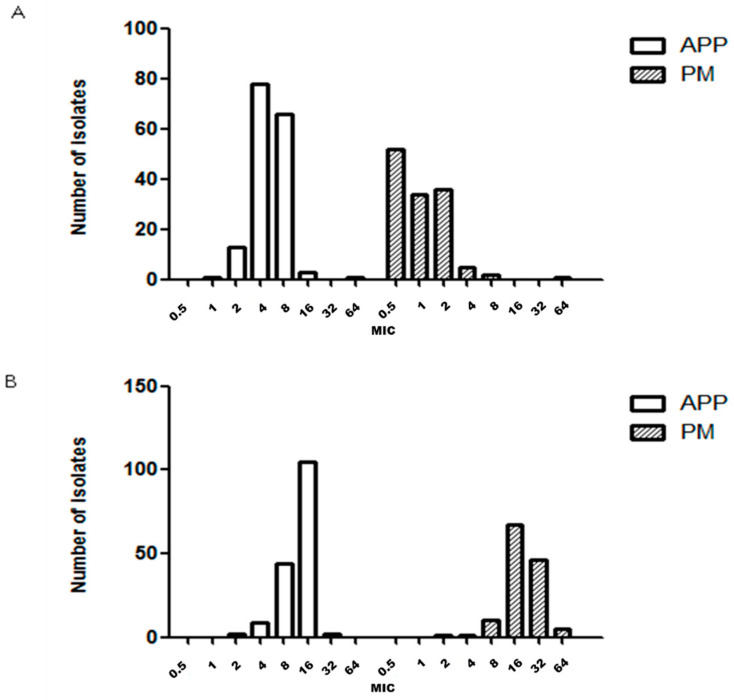
Minimum inhibitory concentration (MIC, μg/mL) distribution of tildipirosin (**A**) and tiamulin (**B**) for *Actinobacillus pleuropneumoniae* (APP) and *Pasteurella multocida* (PM) isolated from lungs of pigs with respiratory symptoms.

**Figure 5:**
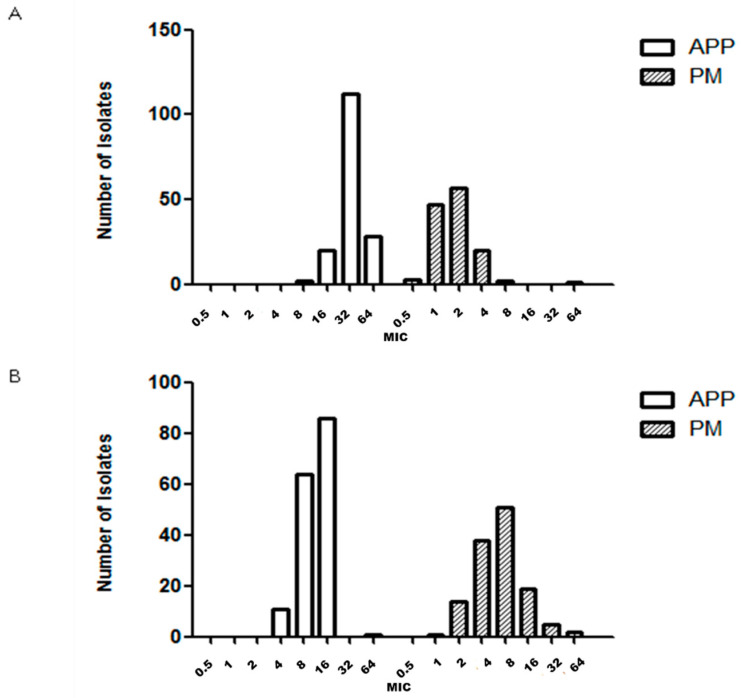
Minimum inhibitory concentration (MIC, μg/mL) distribution of tulathromycin (**A**) and tilmicosin (**B**) for *Actinobacillus pleuropneumoniae* (APP) and *Pasteurella multocida* (PM) isolated from lungs of pigs with respiratory symptoms.

MIC_50_, MIC_90_ and antimicrobial susceptibility for 162, 130 and 29 strains of APP, PM and BB are described in Table 1, Table 2 and Table 3, respectively. The number of GP isolates was low (35 strains) and it was not possible to determine the MIC value because we were unable to grow GP strains with the microdilution technique in our laboratory. The MIC distribution observed for each microorganism and drug are shown in Figure 1, Figure 2, Figure 3, Figure 4 and Figure 5.

APP and PM MIC distributions were very similar for ceftiofur (Figure 1B) and florfenicol (Figure 3A). On the other hand, the APP and PM MIC distributions were different for the following antimicrobials: amoxicillin (Figure 1A), enrofloxacin (Figure 2A), sulfamethoxazole/trimethoprim (Figure 3B), doxycycline (Figure 2B), tildipirosin and tiamulin (Figure 4A,B) and tulathromycin and tilmicosin (Figure 5A,B).

The isolates of APP were highly susceptible (≥90%) to macrolides (tildipirosin, tulathromycin and tilmicosin), tiamulin, florfenicol, sulfamethoxazole/trimethropim and ceftiofur. However, the antimicrobial susceptibility was intermediate (around 72%) for amoxicillin and enrofloxacin and low (35.7%) for doxycycline. *Pasteurella multocida* showed high susceptibility (≥90%) to macrolides (tildipirosin, tulathromycin and tilmicosin), florfenicol, enrofloxacin, amoxicillin and ceftiofur. However, PM antimicrobial susceptibility was intermediate (74.7%) for sulfamethoxazole/trimethropim and tiamulin (60.8%) and low (51.5%) for doxycycline. Thus, in general terms, APP and PM were susceptible to many families of antimicrobials, whereas BB was highly susceptible (100%) only to tildipirosin and tulathromycin. On the other hand, BB susceptibility for florfenicol was close to 50% and <30% for the rest of the antimicrobial families tested (Table 1, Table 2 and Table 3).

## 3. Discussion

There is a scarcity of updated information about antimicrobial susceptibility among porcine pathogens in Spain because there is no official program for their surveillance. The current study aims to address this gap by determining antimicrobial susceptibility, through determining MICs, of ten antimicrobials to three major respiratory tract pathogens recovered, prior to antibiotic treatment, from diseased pigs across Spain. In this case, we have focused on the antimicrobials most frequently used to treat respiratory disease in pigs. 

The measurement of antimicrobial susceptibility is carried out by MIC determination that is more reproducible and comparable between laboratories [11] than disk diffusion techniques due to concerns about disk quality, performance issues [18,19] and variability intrinsically associated to some antimicrobials for the disk diffusion technique [20]. The MIC is the lowest antimicrobial concentration that inhibits in vitro the growth of the target bacteria in specific conditions of in vitro incubation. In this study, the antimicrobial susceptibility has been determined using international guidelines on antimicrobial susceptibility determination [21,22] that cannot be directly compared with other studies only based on disk diffusion techniques [20]. This methodology does not emulate the natural biophase in which bacteria grow in vivo, such as blood, interstitial or intracellular fluid. In any case, antimicrobial sensitivity testing in vitro is used to provide information concerning the efficacy of antimicrobial agents in vivo and thus determine whether an antibiotic is suitable or not to treat a specific condition [23], but MIC determination, as any technique, has weaknesses that should be outweighed [24]. Thus, there is an interesting scientific discussion about the usefulness of MIC to foresee the clinical outcome. Some authors recently have proposed to consider both pathogen- (MIC) and patient-specific drug exposure information to predict treatment success in humans. If both pieces of information are taken into account, it will change how antimicrobials are selected and it will allow optimizing the treatment through precision medicine [25]. Unfortunately, this approach is far away from the “everyday” veterinary medicine, where posology regimens of antimicrobials are fixed for each animal species and bacteria to be treated (registered dose for a veterinary medicinal product). Furthermore, the selection of the antimicrobial is even stricter due to the existence of withdrawal periods in livestock to assure food safety that exclude off-label use of antimicrobials to apply precision medicine [25].

Comparison of antimicrobial susceptibility from other laboratories must be carried out with caution due to inconsistencies in methodology (MIC versus disk diffusion technique), selection of antimicrobial substances in the test panel and variations in interpretation criteria for clinical breakpoints. In our study, the isolates of APP were highly susceptible for macrolides (tildipirosin, tilmicosin and tulathromycin), tiamulin, florfenicol, sulfamethoxazole/trimethropim and ceftiofur. However, the antimicrobial susceptibility was intermediate for amoxicillin and enrofloxacin, and low for doxycycline. This antimicrobial susceptibility pattern described for APP in this study agrees with results obtained by Spanish researchers with strains collected from 1997 [26]. These results could be surprising due to the historic consumption of antimicrobials in Spain and the authors recommend not directly linking the use of antimicrobials with the presence of antimicrobial resistance for any drug–microorganism combination. Moreover, our results are quite similar for isolates from other European countries with some differences [27,28]. Overall, there are still good opportunities to treat infections by APP with antimicrobials, but the presence of strains resistant to doxycycline, amoxicillin and enrofloxacin in Spain highlight the importance of monitoring antimicrobial susceptibility and select the most suitable antimicrobial in a case-by-case situation.

In our study, the isolates of PM were highly susceptible for macrolides (tildipirosin, tilmicosin and tulathromycin), florfenicol, enrofloxacin, amoxicillin and ceftiofur. However, the antimicrobial susceptibility was intermediate for sulfamethoxazole/trimethropim and tiamulin, and low for doxycycline. Thus, the antimicrobial susceptibility pattern is different from that described for APP in spite of the fact that both bacteria are respiratory ones. Again, these results highlight that monitoring of antimicrobial susceptibility must be carried out for each drug–microorganism combination. Moreover, the antimicrobial susceptibility pattern described in our study is very similar to the pattern described by Spanish researchers with strains collected from 1987 [29] for macrolides (>97%), ceftiofur (100%), ampicillin (98%), enrofloxacin (100%), tiamulin (50%) and florfenicol (100%), and by European researchers in a multi-country study to determine the antimicrobial susceptibility of PM in pigs for ceftiofur (100%), enrofloxacin (100%), florfenicol (99.3%), tetracycline (65.8%) and macrolides (>90%) [30]. Thus, the antimicrobial susceptibility of PM seems to have not changed significantly across time, at least, in Europe. This result could be surprising taking into account the enormous variability in the consumption of antimicrobials in livestock across Europe and the authors recommend, again, not directly linking the use of antimicrobials with the presence of antimicrobial resistance for any drug–microorganism combination. 

*Bordetella bronchiseptica* causes a mild or non-progressive inflammation in the nasal cavity that usually needs no treatment. However, if the bacterium is co-infecting with toxigenic PM, it can lead to severe progressive atrophic rhinitis [31]. Moreover, BB may cause pneumonia in young piglets in some cases. In our study, the number of BB strains was quite low to precisely define their MIC distributions. Thus, our results of antimicrobial susceptibility for BB must be interpreted with caution. Moreover, there is a lack of approved clinical breakpoints for many antimicrobials with this bacterium, making comparison with other studies extremely complicated. *Bordetella bronchiseptica* has been described to be intrinsically resistant to ampicillin due to production of beta-lactamases [32,33] and our results agree with this affirmation not only for amoxicillin but also for ceftiofur. In our case, the isolates had extremely high MIC values for doxycycline that exclude them as a therapeutic option to treat BB infection. This lack of antimicrobial susceptibility agrees with Speakman et al. [34] who described a plasmid-encoded tetracycline resistance gene, *tetC*, for this bacterium. Finally, macrolides are also the most susceptible family against BB in Denmark [28], and Dayao et al. [35] also reported no resistance to tulathromycin in Australia.

It is necessary to have valid information about CB to correctly interpret the MIC value obtained in each clinical case. Thus, all the clinical cases with MIC values below CB could be treated with the antimicrobial, at the registered dose, with a high success rate. Unfortunately, there are no clinical veterinary breakpoints available for all the antimicrobials and bacteria for pigs. In our study, we have used well-established CLSI clinical breakpoints for seven out of ten antimicrobials. However, CLSI veterinary breakpoints for sulfamethoxazole/trimethropim and *Pasteurellaceae* have not been set. In this study, the CLSI CB available for *Streptococcus suis* (0.5 μg/mL) have been used. This value exactly agrees with the CB used by El Garch et al. [30] in a study to monitor the antimicrobial susceptibility for sulfamethoxazole/trimethropim of porcine pathogens in Europe, making results directly comparable. These authors carried out this extrapolation due to the high similarity between *Haemophilus influenziae* and respiratory pathogens in pigs. Moreover, The CLSI clinical breakpoint for amoxicillin (0.5 μg/mL) has been obtained from the literature [36]. This CB value for amoxicillin is equal to the CLSI CB for ampicillin that belongs to the same antimicrobial family (beta-lactam antimicrobials). However, Rey et al. [37] proposed that the CB breakpoint (obtained through pharmacokinetics/pharmacodynamic analysis) for amoxicillin, administered by the intramuscular route, could be as low as 0.125 μg/mL for pig respiratory pathogens. If we had used this proposed breakpoint instead of the chosen one (0.5 μg/mL), the percentage of antimicrobial susceptibility for APP and PM would have been 26% and 7% respectively, which is extremely different from the results shown for this antimicrobial in this paper. Thus, there is an urgent need to have CLSI CB breakpoints available for every antimicrobial/bacteria and feedback from swine practitioners when using these antimicrobials at the registered dose [38]. In this sense, the collaboration between microbiologists, pharmacologists and swine practitioners is highly recommended. Finally, CB for doxycycline was extrapolated from CLSI CB available for tetracycline and porcine respiratory pathogens. In general, the percentage of antimicrobial susceptibility determined in our study is comparable with any other study published using CLSI clinical breakpoints. In the case of doxycycline, amoxicillin and sulfamethoxazole/trimethropim, our results must be compared checking the CB used by other authors before making direct comparisons between them. Finally, antimicrobial susceptibility pattern can change with time [28] and this is one of the main reasons to determine it across time in order to select the most suitable antimicrobial, taking into account efficacy criteria and the one-health approach [14].

## 4. Materials and Methods 

### 4.1. Clinical Samples

Samples were drawn from diseased or recently deceased pigs from farms across Spain showing acute clinical signs of respiratory tract infections that had not been exposed to antimicrobial treatment for, at least, 15 days prior to sampling between the years 2017 and 2019. Thus, the sampled animals were between 3 and 24 weeks old, the pigs had overt clinical respiratory signs with or without depression and/or hyperthermia (>39.8 °C) and the mortality rate increased significantly, versus the previous baseline situation, due mainly to respiratory causes at farm level. For each clinical case, samples of lungs of two recently deceased pigs (<12 h) were submitted under refrigeration to the laboratory (to increase possibility to isolation). If, during the veterinary visit, there were no recently dead pigs suitable for sampling, at least two animals with acute respiratory signs were humanely sacrificed and lung samples were drawn. In any case, only one isolate per animal/herd was included in the study. All experimental procedures were approved by the Ethics Committee for Animal Experimentation of the University of Lleida and performed in accordance with authorization 10343 issued by the Catalan Department of Agriculture, Livestock, Fisheries and Food (Section of biodiversity and hunting).

### 4.2. Bacterial Isolation and Identification

Clinical specimens were cultured aseptically onto blood agar (Columbia agar with 5% Sheep blood, 254005 BD), chocolate agar (GC II agar with IsoVitaleX, 254060, BD, Franklin Lakes NJ, USA)) and MacConkey agar (4016702, Biolife Italiana Srl, Milano, Italy) and incubated at 35–37 °C in aerobic conditions with 5–10% CO_2_ for 24–48 hours. Identification of isolates (APP, PM, BB and GP) was carried out by matrix-assisted laser desorption ionization-time of flight (MALDI-TOF Biotyper System, Bruker Daltonics, Bremen, Germany). Individual strains were stored at −80 °C in brain heart infusion (CM1135, Oxoid, Madrid, Spain) with 30% of glycerol (G9012, Sigma-Aldrich, Madrid, Spain). 

### 4.3. Antimicrobial Sensitivity Testing

MIC values were determined using the broth microdilution method by means of customized 96-well microtiter plates (Sensititre, Trek diagnostic Systems Inc., East Grinstead, UK) containing a total of 10 and 7–8 antimicrobials/concentrations respectively, in accordance with the recommendations presented by the Clinical and Laboratory Standards Institute (CLSI) [21,22]. The antimicrobials tested included amoxicillin, ceftiofur, doxycycline, enrofloxacin, florfenicol, sulfamethoxazole/trimethoprim, tiamulin, tilmicosin, tildipirosin and tulathromycin. This antimicrobial panel was selected to represent common compounds licensed for treatment of pig respiratory diseases in practice. 

Bacteria were thawed, cultured on chocolate agar and incubated at 35–37 °C in ambient air (or with 5−10% CO_2_ for APP) for 18–24 h. Three to five colonies were picked and emulsified in demineralized water (or cation-adjusted Mueller–Hinton broth (CAMHB) for APP) to obtain a turbidity of 0.5 McFarland standard (Sensititre™ nephelometer V3011). Suspensions were further diluted in CAMHB (for PM and BB) or Veterinary Fastidious Medium (in the case of APP) to reach a final inoculum concentration of 5 × 10^5^ colony forming units (cfu)/mL (Table 4). Then, the Sensititre panel was reconstituted by adding 100 μL/well of the inoculum. Plates containing PM and BB isolates were incubated at 35 ± 2 °C for 18−20 h. In the case of APP isolates, plates were covered with a perforated seal and incubated at 35 ± 2 °C, with 5−10% CO_2_ for 20–24 h [21,22]. The antibiotic panels were read manually using Sensititre™ Vizion (V2021) and the MIC value was established as the lowest drug concentration inhibiting visible growth. For each strain tested, a colony count and a purity check were performed following CLSI and manufacturer recommendations. Moreover, quality control strains were also included in all susceptibility testing runs. Thus, *Actinobacillus pleuropneumoniae* (ATCC 27090™) and *Escherichia coli* (ATCC 25922™) were included as quality control [21,22]. The MICs of the quality control strains had to be within acceptable CLSI ranges to accept the results obtained in the laboratory. 

### 4.4. Data Analysis

All the clinical cases with MIC values below CB were classified as susceptible because they could be treated with the antimicrobial, at the registered dose, with a high success rate. The results of the sensitivity tests are presented as MIC distributions and these were determined for each species–antimicrobial combination. MIC_50_ and MIC_90_ were defined as MICs inhibiting 50% and 90% of the strains, respectively. Clinical breakpoints from CLSI were used [21,22] to determine antimicrobial susceptibility. However, CLSI veterinary breakpoints for sulfamethoxazole/trimethropim and *Pasteurellaceae* have not been set. Thus, the CLSI CB available for *Streptococcus suis* (0.5 μg/mL) and sulfamethoxazole/trimethropim have been used in this study. The clinical breakpoint for amoxicillin (0.5 μg/mL) has been obtained from the literature [36] and CLSI CB available for tetracycline (0.5 μg/mL) and porcine respiratory pathogens was extrapolated for doxycycline [21,22]. The antimicrobial susceptibility was considered high at levels ≥90% and low at levels ≤60%, as described by Holmer et al. [28]. 

## 5. Conclusions

The obtained antimicrobial susceptibility varies between pig respiratory pathogens. *Actinobacillus pleuropneumoniae* and *Pasteurella multocida* were highly susceptible to ceftiofur, florfenicol and macrolides. However, the antimicrobial susceptibility was intermediate for amoxicillin and enrofloxacin in the case of APP and sulfamethoxazole/trimethropim and tiamulin in the case of PM. Both bacteria showed low antimicrobial susceptibility to doxycycline. Finally, *Bordetella bronchiseptica* was highly susceptible only to tildipirosin and tulathromycin and its susceptibility for florfenicol was close to 50% and <30% for the rest of the antimicrobial families tested. These results emphasize the need for determining antimicrobial susceptibility in pig respiratory cases in order to optimize the antimicrobial treatment in a case-by-case scenario and provide a robust criteria to select the most suitable antimicrobials, taking into account the one-health approach. On the other hand, there is an urgent need to have CLSI CB breakpoints available for every antimicrobial/bacteria and feedback from swine practitioners when using these antimicrobials at the registered dose.

## Figures and Tables

**Table 1:** *Actinobacillus pleuropneumoniae* (APP), MIC_50_, MIC_90_, current recommended clinical breakpoints (CB) and antimicrobial susceptibility. The MIC_50_ and MIC_90_ were determined from the MIC distribution from 162 APP strains isolated from respiratory clinical cases. The antimicrobial susceptibility was calculated as the percentage of bacterial isolates below CB.

Antimicrobial	MIC_50_ (μg/mL)	MIC_90_ (μg/mL)	Clinical Breakpoint (CB) ^1^ (μg/mL)	Antimicrobial Susceptibility Based on CB
Amoxicillin	0.25	16	0.5 ^$^	72.2
Ceftiofur	0.06	0.06	2	100
Doxycycline	1	4	0.5 ^+^	35.7
Enrofloxacin	0.06	1	0.25	72.2
Florfenicol	0.25	0.25	2	97.0
Sulfamethoxazole/trimethropim ^&^	0.125	2	0.5	88.9
Tiamulin	16	16	16	98.8
Tildipirosin	4	8	16	99.4
Tilmicosin	8	16	16	99.4
Tulathromycin	32	64	64	100

^1^ All clinical breakpoints were obtained from Clinical and Laboratory Standards Institute (CLSI) M100 2018 and CLSI VETO8 4th ed., 2018, with the following clarifications: ^$^ Schwarz et al. (2008) [36]. ^+^ Extrapolated from tetracycline. ^&^ MIC represented in the table is for trimethropin. Sulfamethoxazole/trimethropim ratio tested is 19:1.

**Table 2:** *Pasteurella multocida* (PM), MIC_50_, MIC_90_, current recommended clinical breakpoints (CB) and antimicrobial susceptibility. The MIC_50_ and MIC_90_ were determined from the MIC distribution from 130 PM strains isolated from respiratory clinical cases. The antimicrobial susceptibility was calculated as the percentage of bacterial isolates below CB.

Antimicrobial	MIC_50_ (μg/mL)	MIC_90_ (μg/mL)	Clinical Breakpoint (CB) ^1^ (μg/mL)	Antimicrobial Susceptibility Based On CB
Amoxicillin	0.25	0.5	0.5 ^$^	96.2
Ceftiofur	0.06	0.12	2	100
Doxycycline	0.5	4	0.5 ^+^	51.5
Enrofloxacin	0.03	0.06	0.25	98.5
Florfenicol	0.5	0.5	2	100
Sulfamethoxazole/trimethropim ^&^	0.12	4	0.5	74.7
Tiamulin	16	32	16	60.8
Tildipirosin	1	2	4	97.7
Tilmicosin	8	16	16	94.6
Tulathromycin	2	4	16	100

^1^ All clinical breakpoints were obtained from CLSI M100 2018 and CLSI VETO8 4th ed., 2018 with the following clarifications: ^$^ Schwarz et al. (2008) [36]. ^+^ Extrapolated from tetracycline. ^&^ MIC represented in the table is for trimethropin. Sulfamethoxazole/trimethropim ratio tested is 19:1.

**Table 3:** *Bordetella bronchiseptica* (BB), MIC_50_, MIC_90_, current recommended clinical breakpoints (CB) and antimicrobial susceptibility. The MIC_50_ and MIC_90_ were determined from the MIC distribution from 29 BB strains isolated from respiratory clinical cases. The antimicrobial susceptibility was calculated as the percentage of bacterial isolates below CB.

Antimicrobial	MIC_50_ (μg/mL)	MIC_90_ (μg/mL)	Clinical Breakpoint (CB) ^1^ (μg/mL)	Antimicrobial Susceptibility Based On CB
Amoxicillin	16	16	0.5	0
Ceftiofur	4	4	2	0
Doxycycline	1	2	0.5	27.7
Enrofloxacin	0.5	0.5	0.25	20.7
Florfenicol	2	4	2	51.7
Sulfamethoxazole/trimethropim ^&^	4	8	0.5	3.4
Tiamulin	64	64	16	0
Tildipirosin	4	8	8	100
Tilmicosin	32	64	16	27.6
Tulathromycin	8	8	16	100

^1^ Florfenicol, tildipirosin and tulathromycin CB were obtained from CLSI M100 2018 and CLSI VETO8 4th ed., 2018. The rest of the CB were extrapolated from *Pasteurella multocida* (Table 2). ^&^ MIC represented in the table is for trimethropin. Sulfamethoxazole/trimethropim ratio tested is 19:1.

**Table 4:** Details of the conditions used to carry out minimum inhibitory concentration (MIC) determination using the broth microdilution method by means of customized 96-well microtiter plates (Sensititre, Trek diagnostic Systems Inc., East Grinstead, UK).

Microorganism	0.5 McFarland Suspension Medium	Broth	Final Inoculum	Plate Reconstitution	Incubation Conditions
*Pasteurella multocida* and *Bordetella bronchiseptica*	Water	CAMHB	5 × 10^5^ cfu/mL	100 μL	35 ± 2 °C 18−24 h Non-CO_2_ incubator
*Actinobacillus pleuropneumoniae*	CAMHB	VFM	5 × 10^5^ cfu/mL	100 μL	35 ± 2 °C 20−24 h CO_2_ incubator perforated seal

CAMHB—Cation-adjusted Mueller–Hinton Broth. VFM—Veterinary Fastious Medium. cfu—colony forming units.
